# Ecometrics demonstrates that the functional dental traits of carnivoran communities are filtered by climate

**DOI:** 10.1002/ece3.70214

**Published:** 2024-10-13

**Authors:** Leila Siciliano‐Martina, Jenny L. McGuire, Maria A. Hurtado‐Materon, Rachel A. Short, Daniel A. Lauer, Julia A. Schap, Johannes Müller, Fredrick K. Manthi, Jason J. Head, A. Michelle Lawing

**Affiliations:** ^1^ Department of Biology Texas State University San Marcos Texas USA; ^2^ Department of Ecology and Conservation Biology Texas A&M University College Station Texas USA; ^3^ School of Biological Sciences, Georgia Institute of Technology Atlanta Georgia USA; ^4^ School of Earth and Atmospheric Sciences, Georgia Institute of Technology Atlanta Georgia USA; ^5^ Interdisciplinary Graduate Program in Quantitative Biosciences Georgia Institute of Technology Atlanta Georgia USA; ^6^ Ecology and Evolutionary Biology Program Texas A&M University College Station Texas USA; ^7^ Department of Natural Resource Management South Dakota State University Rapid City South Dakota USA; ^8^ Museum für Naturkunde, Leibniz‐Institut für Evolutions‐Und Biodiversitätsforschung Berlin Germany; ^9^ Department of Earth Sciences National Museums of Kenya Nairobi Kenya; ^10^ Department of Zoology University of Cambridge Cambridge UK; ^11^ University Museum of Zoology, University of Cambridge Cambridge UK

**Keywords:** carnassial teeth, carnivorans, dietary ecology, ecometrics, paleoclimate

## Abstract

Terrestrial carnivorans, with their diverse diets and unique adaptations such as the carnassial tooth, offer insights into the connections between functional traits and the climatic and environmental conditions they inhabit. They shed light on functional trait‐environment relationships at the highest trophic levels across a broad range of environmental conditions. In this study, we evaluate the relationship between relative blade length (RBL) of the lower carnassial tooth, a key dietary adaptation among terrestrial carnivorans for slicing and grinding food items, and climate. We propose RBL as an ecometric trait and test the hypothesis that community‐level RBL is correlated with climate and mediated by environmental effects on food availability. Our findings show that communities with higher mean and broader variance of RBL are typically located in warmer and wetter climates, suggesting a relationship between carnivoran dietary diversity and climate. Conversely, communities with a lower mean and narrower variance of RBL predominantly occupy cooler, drier places. This indicates that community‐level carnivoran dietary traits have the potential to serve as indicators of environmental conditions. Given the robust fossil record associated with carnivorans, we also show how RBL can be used as a proxy for reconstructing paleoclimates by examining trait change at seven sites in North America to estimate changes in temperature and precipitation over time in relation to changes in carnivoran community assembly. Understanding the nature of trait‐environment relationships can help us anticipate biological impacts of ongoing environmental change and the geographic regions at the greatest risk of ecological disruption.

## Graphical abstract

Carnivorans can provide a unique insight into the relationship between functional dietary traits and environmental conditions. Using ecometric analyses, we evaluated the relationship between the relative blade length of the carnivoran carnassial tooth and temperature and precipitation at global and continental scales. We detected a relationship in which greater trait variance is associated with warmer, wetter conditions, suggesting a relationship between carnivoran dietary diversity and climate.

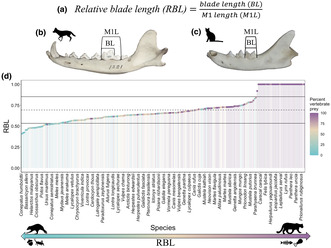

## 1 INTRODUCTION

Understanding the factors that guide community assembly and global habitation patterns is a perennial challenge in biogeography, ecology, and conservation research (Gaston, 2000; Mittelbach & Schemske, 2015; Weiher et al., 2011). Communities are often sorted geographically across temperature and precipitation gradients, patterns that are mediated by the functional traits displayed by community members (Violle et al., 2014; Weiher et al., 2011; Wiens, 2011). Functional traits describe attributes that are strongly related to organismal performance and may include a variety of features such as dental morphology, locomotor strategy, and behavioral and physiological attributes (Eronen, Polly, et al., 2010; McGill et al., 2006). As we develop an understanding of the relationships between functional traits and environments, we can identify the geographic regions that may be at risk of future disruptions to those relationships, and we can understand how ecometric relationships may be modulated both spatially and temporally (McGuire et al., 2023).

Ecometrics evaluate trait‐environment relationships at the community level across spatial gradients, thereby accounting for the trait diversity of a functional assemblage (Eronen, Polly, et al., 2010; Polly et al., 2011; Polly & Head, 2015; Vermillion et al., 2018). Ecometric traits that represent dietary ecology, locomotory strategy, and physiology have been identified in several groups of vertebrates (Barr, 2017; Gruwier & Kovarovic, 2022; Head et al., 2009; Meloro & Sansalone, 2022; Oksanen et al., 2019; Polly, 2010; Short & Lawing, 2021). For example, the locomotor traits of carnivoran communities are indicative of ecoregion and vegetation type (Polly, 2010; Short et al., 2023). Carnivoran communities composed of cursorial species with digitigrade hindlimb traits are frequently located in more open, grassland habitats, whereas plantigrade communities, more commonly associated with an arboreal strategy, are frequently located in woodland habitats (Polly, 2010, 2020). Ecometrics can provide robust models of trait‐environment relationships across global ecosystems (Eronen, Puolamäki, et al., 2010; Short et al., 2023; Short & Lawing, 2021) and can be used to hindcast environmental conditions associated with paleocommunities (Schap et al., 2021; Schap et al., 2024; Short et al., 2023; Short & Lawing, 2021). Given that carnivorans are geographically widespread, inhabit diverse environmental conditions, and have highly variable diets, the trait‐environment relationships associated with their dietary morphology may illuminate environmental factors that have yet to be adequately addressed by exploring other trophic groups. Carnivorans are also represented by an extensive paleontological record, which provides a further opportunity to explore these trait‐environment relationships across a temporal scale.

Dietary ecometrics are well developed for herbivorous species, including the tooth crown heights and loph counts of ungulates in relationship to precipitation (e.g., Eronen et al., 2010b; Fortelius et al., 2002, 2014; Jernvall & Fortelius, 2002; Žliobaite et al., 2018), the loph counts of ungulates and primates in relationship to temperature (Oksanen et al., 2019), the tooth crown heights of small mammals in relationship to temperature and precipitation (e.g., Schap et al., 2021; Schap et al., 2024), and the combined tooth crown heights of small and large herbivorous mammal species in relationship to precipitation (Short et al., 2021). Given that herbivorous diets are directly related to primary productivity, these communities are expected to display a tight ecometric relationship between trait morphology and environmental conditions. Terrestrial carnivorans display wide dietary preferences, from strict obligate carnivores (e.g., felids) to predominantly herbivorous species (e.g., giant panda, *Ailuropoda melanoleuca*) as well as an array of omnivorous, frugivorous, and invertivorous species (Friscia et al., 2007; Middleton et al., 2021; Van Valkenburgh, 1989). Terrestrial carnivoran diets are also expected to be indirectly related to the environment through their foods, where the availability of fruits and seeds, as well as vertebrate and invertebrate prey are also strongly dictated by climatic conditions (Cruz et al., 2022; Meloro, 2022; Van Valkenburgh, 1989). Across geographic regions, terrestrial carnivoran communities are known to display an increase in trophic diversity in habitats characterized by higher temperatures and precipitation given the increased biodiversity and ample availability of dietary options in those areas, including fruits, seeds, nuts, and a diverse array of invertebrates (Cruz et al., 2022; Ray & Sunquist, 2001; Zhou et al., 2011; Zuercher et al., 2022). We may therefore expect a biogeographic trend in which the dietary breadth of a carnivoran community is reflective of dietary opportunity; however, carnivoran dietary traits have yet to be explored in an ecometric framework.

Carnassial teeth are an identifying feature of terrestrial carnivoran dentition and include the first mandibular molar (m1) and the fourth cranial premolar (P4) (Friscia et al., 2007; Tarquini et al., 2020; Van Valkenburgh, 2007). The lower carnassial is composed of two primary regions: the trigonid blade, which is instrumental in gnashing and tearing, and the talonid basin, which is involved in grinding and crushing food items (Friscia et al., 2007; Tarquini et al., 2020; Van Valkenburgh, 1989). Carnassial teeth can display considerable morphological variation associated with the dietary niche of the species (Friscia et al., 2007; Tarquini et al., 2020; Van Valkenburgh, 1989). The relative blade length (RBL) of the m1 carnassial tooth has been widely used to assess carnivoran diets and is an easily recognizable trait that is frequently preserved in both modern and fossil specimens (Balisi et al., 2018; Friscia et al., 2007; Holliday & Steppan, 2004; Van Valkenburgh, 1989). The RBL measure assesses the length of the trigonid blade relative to the overall m1 tooth length, thereby representing the proportion of the tooth devoted to the cutting blade. The RBL essentially represents the degree to which the carnassial tooth is modified for a carnivorous diet, signified by a well‐developed trigonid blade, or a less carnivorous diet with a larger area devoted to the talonid basin (Davies et al., 2007; Friscia et al., 2007; Van Valkenburgh, 1989). RBL varies across Carnivora from frugivorous species, such as dwarf mountain coatis (*Nasuella olivacea*) who have a minimal trigonid blade and a relatively large talonid basin with an RBL value of 0.47 (Friscia et al., 2007), to obligate carnivores, such as the mountain lion (*Puma concolor*) which, like all felids, have carnassial teeth that are entirely composed of the trigonid blade with an RBL value of 1.0 (Van Valkenburgh, 1989).

Carnivoran communities vary geographically due to ecological and evolutionary processes implicated in community assembly; therefore, large geographic regions, such as continents, may be associated with differing ecometric patterns. Over the past few centuries, carnivorans have also experienced massive shifts in their geographic ranges due to habitat loss, expansion of urban areas, purposeful extermination, and other anthropogenic disruptions, factors which have disproportionately influenced large hypercarnivorous species, due in part to their extensive habitat requirements (Di Minin et al., 2016; Fernández‐Sepúlveda & Martín, 2022; Ripple et al., 2014). Large carnivorans perform essential ecological roles and are often characterized as keystone species; therefore, the eradication of these animals from community assemblages can have severe consequences for ecosystem function (Gittleman & Gompper, 2005; Ripple et al., 2014; Sih et al., 1998). The loss of large carnivorans from community assemblages is also known to trigger an increase in mesocarnivorous species inhabiting an area (Brashares et al., 2010; Hoeks et al., 2020), thereby altering the community‐wide trait distributions. These changes to carnivoran community assemblages have been experienced more acutely in certain global regions associated with heightened levels of agriculture and human population density (Ripple et al., 2014; Wolf & Ripple, 2017). For example, large carnivorans have been fully extirpated in regions of the eastern United States and Europe and are at heightened risk of extinction in southeast Asia (Dalerum et al., 2009; Wolf & Ripple, 2017). Certain mid‐sized carnivorans (e.g., red fox, *Vulpes vulpes*; common raccoon, *Procyon lotor*) have expanded their geographic ranges in these regions (Bateman & Fleming, 2012) and changed the overall structure of these carnivoran assemblages. Therefore, turnover in carnivoran community assemblages can be reflective of climatic conditions as well as human disruption.

Here, we leverage one aspect of terrestrial carnivoran dental morphology, namely carnassial relative blade length (hereafter RBL), to test whether there is a trait‐environment relationship at the community level by developing ecometric models of temperature and precipitation. We expected regions with higher precipitation to support greater availability and variety of dietary items and therefore to be associated with a greater variation in community‐wide dietary traits, whereas we expected regions with low precipitation to support fewer diet types and to have reduced trait variation with mean trait values primarily associated with carnivorous behaviors. Additionally, we expected that temperature may be related to community‐level dietary trait values in carnivorans because prey composition and prey environment vary with temperature. We calibrated ecometric models for each continent to investigate whether ecometric relationships hold across different large geographic regions that have different climate compositions and that have also recently experienced different amounts of carnivoran loss. We expected the strongest overall relationships to be associated with regions with the most variation in precipitation (e.g., Asia and South America) and temperature (e.g., Asia and North America) and the weakest relationships to be associated with areas with greater habitat loss (e.g., Europe). Finally, we evaluated functional trait shifts associated with seven North American paleocommunities to assess whether this relationship holds through time despite carnivoran extirpations and extinctions. We expected paleocommunities associated with drier conditions to display less variation in dietary traits and to have mean trait values more strongly associated with carnivorous dietary habits as compared to modern communities found in the same localities in wetter conditions, which may have a greater diversity of dietary options.

## 2 METHODS

### 2.1 Study system and functional trait

Carnivorans represent an ecologically diverse mammalian order of 252 extant terrestrial species (IUCN, 2020). Terrestrial carnivorans are native to five continents worldwide (apart from Antarctica and Australia) and can be found in nearly every global ecoregion across a range of temperature and precipitation gradients (Arias‐Alzate et al., 2020; Van Valkenburgh, 2007; Wilson & Mittermeier, 2009). Whereas certain species may be highly adapted for life in extreme habitats such as deserts (e.g., fennec fox, *Vulpes zerda*, sand cat, *Felis margarita*) or tundras (e.g., Canada lynx, *Lynx canadensis*, polar bear, *Ursus maritimus*) (Ripple et al., 2014; Wilson & Mittermeier, 2009), some carnivorans display generalized habitat requirements and may flourish in any number of ecosystems including human‐dominated landscapes (e.g., red fox, *Vulpes vulpes*, raccoon, *Procyon lotor*; Bateman & Fleming, 2012; Gehrt et al., 2010). Carnivorans also perform a variety of roles within their communities that help to maintain ecosystem function, including as large apex predators that act as top‐down community regulators as well as smaller mesocarnivores that act as predators, competitors, and seed dispersers (Dalerum, 2013; Prevosti & Pereira, 2014; Roemer et al., 2009).

**FIGURE 1:**
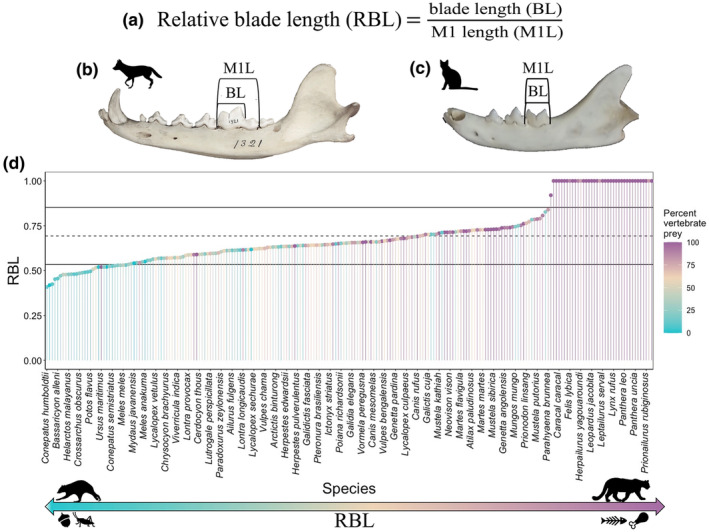
The carnassial relative blade length (RBL) trait. (a) The RBL calculation is based on the ratio of the trigonid blade length (BL) divided by the total length of the m1 tooth (m1L). (b) Carnivorans with diets that extend beyond vertebrate prey, such as coyotes (*Canis latrans*), have a smaller relative blade length. (c) Obligate carnivores, such as cats, have an m1 composed exclusively of the trigonid blade. (d) Rank‐order plot showing the RBL values of the species in our dataset color‐coded based on the percent of vertebrate prey associated with the species in Elton Traits 1.0 (Wilman et al., 2014). Diets with a higher proportion of vertebrate prey tend to have higher RBL values.

To assess the functional dietary traits of terrestrial carnivorans, we compiled the relative blade lengths (RBL) of the mandibular m1 tooth for 223 of 252 non‐domesticated extant terrestrial carnivoran species as reported by the IUCN (IUCN, 2020) (Figure 1c). These measures were recorded from the existing published literature as well as from direct measurements (using complete mandibles) collected within the National Museums of Kenya (Nairobi, Kenya) using digital calipers. In some cases, we also measured the RBL from specimen photos with Fiji software version 2.3.0 (Schindelin et al., 2012) using specimen images from natural history collections that were published in the literature (e.g., Christiansen, 2009; Larivière, 2001; Morales & Giannini, 2010, Nellis, 1989; Tellaeche et al., 2018; Van Rompaey, 1988; Van Rompaey & Colyn, 1992; *n* = 7), had been previously photographed in a museum collection (*n* = 3), or were available online through museum collections (e.g., Animal Diversity Web; Myers et al., 2024; *n* = 2) (Appendix S1). When possible, measures were collected from a minimum of five specimens (including males and females) to calculate the mean RBL for each species. We did not measure any specimens with damaged or broken carnassial teeth or specimens from immature individuals (assessed using cranial sutures and tooth eruption patterns).

### 2.2 Ecomorphological analyses

Previous work has documented an ecomorphological signal in the trait variation of RBL (Friscia et al., 2007; Tarquini et al., 2020; Van Valkenburgh, 1989). As a preliminary analysis, we assessed whether that signal is present in the dataset we assembled to test whether biological processes are drivers of our findings rather than statistical artifacts. We tested whether RBL variance is explained by two aspects of the carnivoran diet: the percent of vertebrate prey items, as reported in Elton Traits 1.0 (Wilman et al., 2014), and dietary diversity. To estimate dietary diversity, we calculated the number of diet categories that composed at least 10% of the diet of each species including vertebrate prey, invertebrate prey, fruits, plant material, seeds, and scavenged materials (following Wilman et al., 2014). To evaluate the relationship between RBL, percent vertebrate prey, and dietary diversity, we conducted Phylogenetic Generalized Least Squares (PGLS) using the carnivoran phylogeny from Nyakatura and Bininda‐Emonds (2012).

### 2.3 Community‐level data

We assembled species lists by overlaying IUCN range maps (IUCN, 2020) with a set of points sampled equidistantly at 50 km across the terrestrial globe (excluding Antarctica and Australia) resulting in 50,994 community sampling points (Polly, 2010). We limited our dataset to areas with a minimum of three carnivoran species with RBL trait measures, which left us with 48,757 global communities, including 11,835 communities in Africa, 17,162 communities in Asia, 3817 communities in Europe, 8948 communities in North America, and 6995 communities in South America. Any taxonomic discrepancies between the published literature, the ASM Mammal Diversity Database (Mammal Diversity Database, 2023), and the IUCN range maps (IUCN, 2020), were resolved based on the Integrated Taxonomic Information System (ITIS, 2023).

To test whether there is a relationship between community‐level RBL traits and climate, we sampled annual precipitation (AP, mm) and mean annual temperature (MAT, C) at the geographic location of every community in our dataset from interpolated global environmental data sourced from weather stations at a resolution of 1 km^2^ (Fick & Hijmans, 2017). We log transformed annual precipitation because ecologically relevant variation in small precipitation values is underemphasized in standard deviation calculations compared to large precipitation values.

### 2.4 Ecometric analyses

We calculated the mean and standard deviation of the RBL trait for each community within our dataset and organized those data into a 25 × 25 gridded ecometric space (625 ecometric bins total) following Lawing & Polly (2012) and Vermillion et al. (2018). We used a maximum likelihood framework to separately fit trait models with AP and MAT (Figure 4). This method has been shown to produce more accurate estimates than other statistical approaches to fitting ecometric models (Short et al., 2021). For each individual ecometric bin, we created a likelihood surface using a Gaussian kernel smoother to estimate the distribution and fit of the environmental values of all the communities within the bin. We then extracted the most likely environmental value from the peak of the likelihood surface. We evaluated the fit of the trait‐environment relationship by comparing the estimated environmental values to the observed environmental values by calculating the coefficient of determination (*R* ^2^).

To test whether the estimated to observed environment correlation is spurious, we shuffled the estimated environments and compared those environments to the observed environments with the coefficient of determination. For a second test, we randomly assigned communities to an ecometric bin, recalculated the maximum likelihood value of the environment for all of the newly assigned communities within the bin, and compared those estimates to the observed values with a coefficient of determination. We repeated both procedures 100 times and compared the distribution of correlations generated from the randomization procedures with the original correlation between the estimated and observed environments. In addition, we calculated the anomaly between the observed and estimated environments for every community and mapped the anomalies in geographic space to investigate spatial patterning. We conducted this procedure for all communities globally and repeated these steps for the communities associated with each continent.

We assessed the validity and transferability of the RBL ecometric model with a series of sensitivity analyses following Schap et al. (2024). We randomly down‐sampled communities with sample sizes ranging from 100 to 9100 communities at intervals of 1000 to test various sample sizes across the full range of sample sizes while limiting the total number of required iterations, and we repeated this procedure 20 times to obtain a range of variation associated with the different sample sizes. For each down sample, we used a randomly drawn 80% of the data to train an ecometric model and the remaining 20% for testing. The final RBL ecometrics model was fit using all communities, which is above the number of communities needed to accurately capture the RBL trait‐environment relationship.

### 2.5 Paleoenvironmental reconstruction

We used the carnivoran RBL trait to reconstruct paleoenvironmental conditions and assessed the trends associated with seven North American fossil sites from the Last Glacial Maximum (~40,000 to 10,000 years ago). These sites included Anderson Pit, Indiana (Richards, 1972), Brynjulfson Cave 1, Missouri (Parmalee & Oesch, 1972), Friesenhahn Cave, Texas (Graham, 1976), January Cave, Alberta (Burns, 1991), Little Box Elder Cave, Wyoming (Anderson, 1968; Long, 1971), McKittrick, California (Jefferson, 1991), and New Trout Cave, West Virginia (Grady, 1986). The carnivoran species associated with these sites were extracted from the Neotoma Paleoecology Database (https://www.neotomadb.org) and were previously used in ecometric studies by Polly et al. (2017). Using these species lists, we compiled RBL values for the assemblages reported from each fossil site. We compiled additional RBL measures for extinct species (*n* = 3) by measuring specimen images from the published literature (Christiansen & Harris, 2009; Reynolds et al., 2023; Sorkin, 2006).

To reconstruct the paleoenvironments associated with the fossil sites, we calculated the mean and standard deviation of the RBL trait for each fossil community. We used the means and standard deviations from the fossil sites to explore the temperature and precipitation estimates of these areas based on the global ecometric models fitted using the modern carnivoran communities (Polly & Head, 2015; Schap et al., 2021; Schap et al., 2024; Short et al., 2021, 2023). To determine how community trait distributions temporally shifted, we extracted mean and standard deviation values of the modern carnivoran communities currently inhabiting the locations associated with each fossil site. Unless otherwise noted, all analyses were conducted in R version 4.2.3 (R Core Team, 2023).

## 3 RESULTS

### 3.1 Relative blade length

**FIGURE 2:**
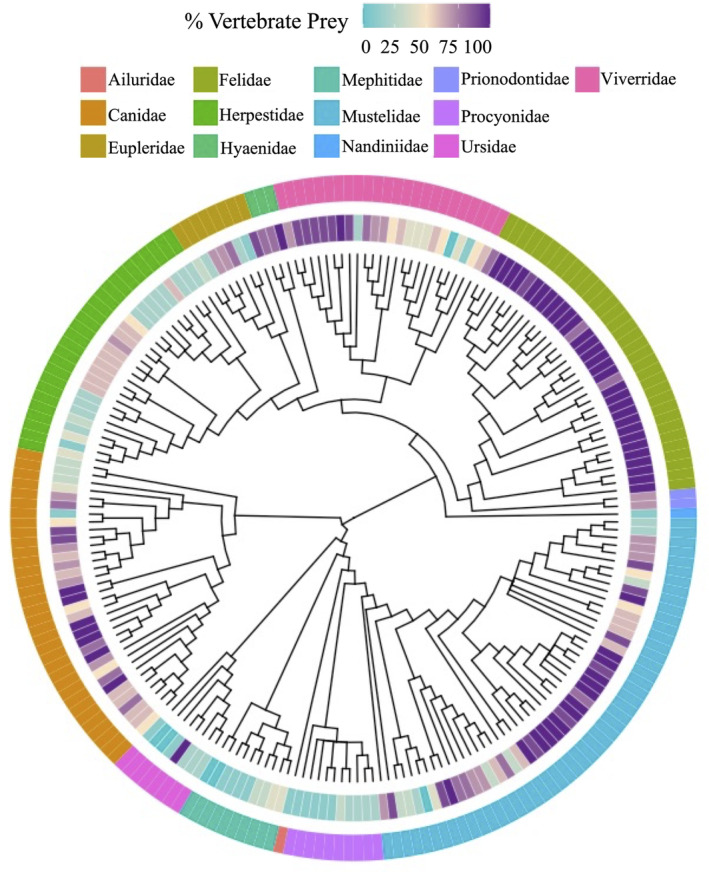
Phylogeny of carnivorans included in the dataset, pruned from Nyakatura and Bininda‐Emonds (2012). Tree tips are color‐coded based on the percentage of vertebrate prey in the species diets as reported in Elton Traits 1.0 (Wilman et al., 2014). The outer ring indicates the taxonomic families represented on the phylogeny.

Relative blade lengths varied across species from 0.41 for Humboldt's hog‐nosed skunk (*Conepatus humboldtii*) to 1.0 for the felids (Figure 1). This variation was associated with diet, where higher RBL values are associated with a higher percent of vertebrate prey in the diet (Figure 1d). Although RBL has a high phylogenetic signal (Pagel's lambda = 0.97) (Figure 2), PGLS analyses indicate that 14% of the variation in species‐level RBL is explained by both percent vertebrate prey in the diet (*R* ^2^ = .14; *p* = .02) and dietary diversity (*R* ^2^ = .14; *p* = .03).

### 3.2 Geographic trait variation

**FIGURE 3:**
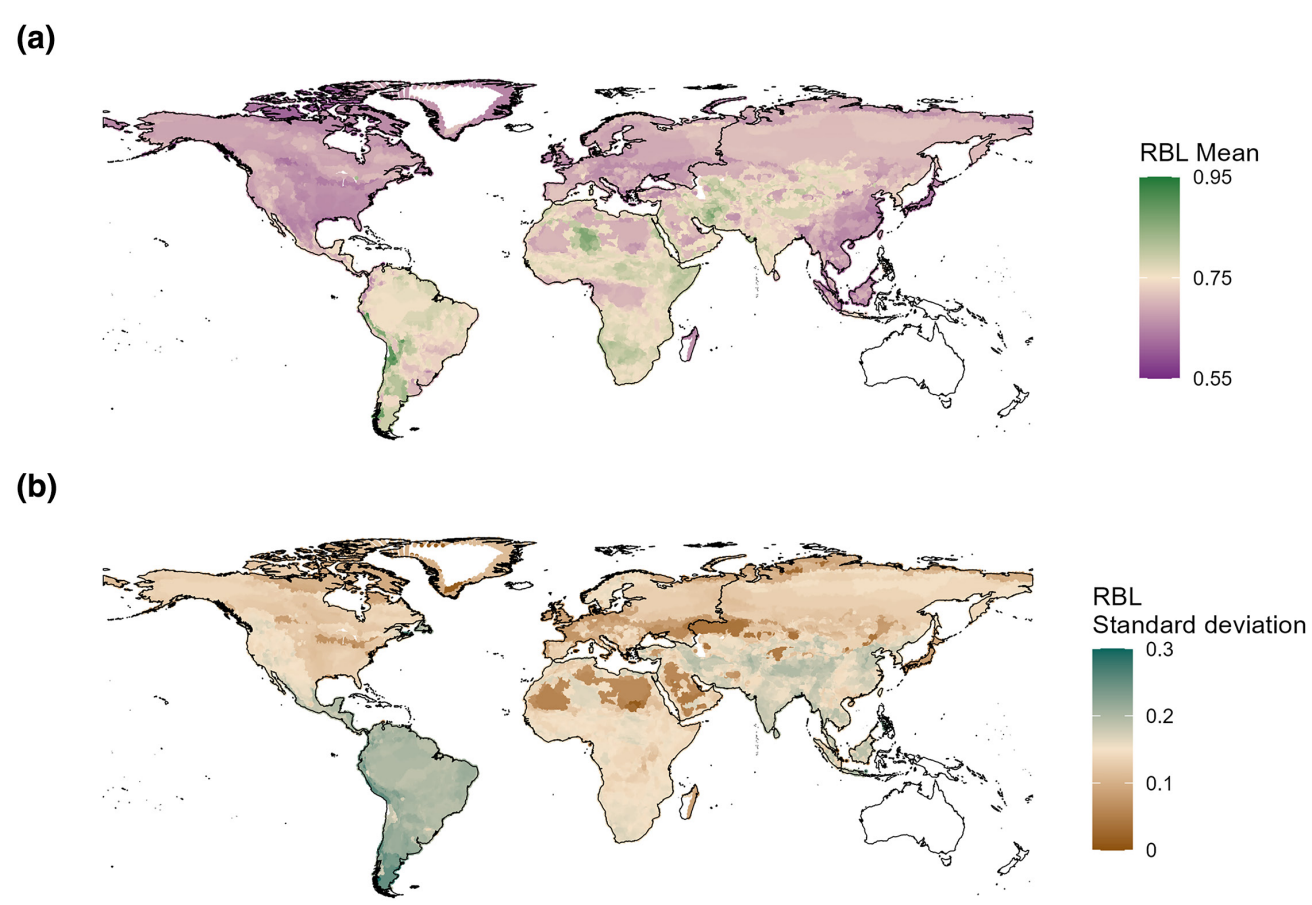
Geography of carnivoran community RBL traits. Maps were created by sampling carnivoran communities composed of a minimum of three species traits at 50‐km point intervals. Areas in white indicate regions that have fewer than three carnivoran species per community. (a) Mean carnivoran community RBL. (b) Standard deviation in carnivoran community RBL.

Community mean RBL ranged from 0.53 to 0.94 worldwide with a standard deviation of 0.15 (Table 1), where the highest values were most apparent in arid regions, such as the Atacama Desert of western South America, the central Sahara Desert of Africa, and the desert region of southwestern and central Asia (Figure 3a). Low mean RBL values were found throughout most of the Holarctic region of North America and Europe as well as northern and eastern Asia (Figure 3a). Certain geographic regions were also associated with a high degree of within‐community trait variation, including tropical regions, such as most of South and Central America as well as portions of southeast Asia (Figure 3b). Low trait variation was noted in desert regions of northern Africa and portions of western Asia (Figure 3b).

**TABLE 1:** Results of the ecometric models.

Localities	*N*	Min RBL	Max RBL	Mean RBL (*σ*)	*R* ^2^ (MAT)	*R* ^2^ (AP)
Global	48,757	0.53	0.94	0.72 (0.15)	.53	.49
Africa	11,835	0.64	0.88	0.75 (0.14)	.10	.59
Asia	17,162	0.55	0.87	0.72 (0.14)	.52	.66
Europe	3817	0.63	0.79	0.69 (0.11)	.36	.46
North America	8948	0.53	0.83	0.68 (0.13)	.71	.45
South America	6995	0.61	0.94	0.76 (0.20)	.67	.64

*Note*: For each geographic region, we are reporting the number of carnivoran communities (*N*) that include a minimum of three species traits, as well as the minimum (min), maximum (max), mean, and standard deviation (*σ*) of the RBL trait for each region and the *R* ^2^ associated with the observed to expected climatic models for mean annual temperature (MAT) in Celsius (C) and annual precipitation (AP) in millimeters (mm).

### 3.3 Modern carnivoran communities

**FIGURE 4:**
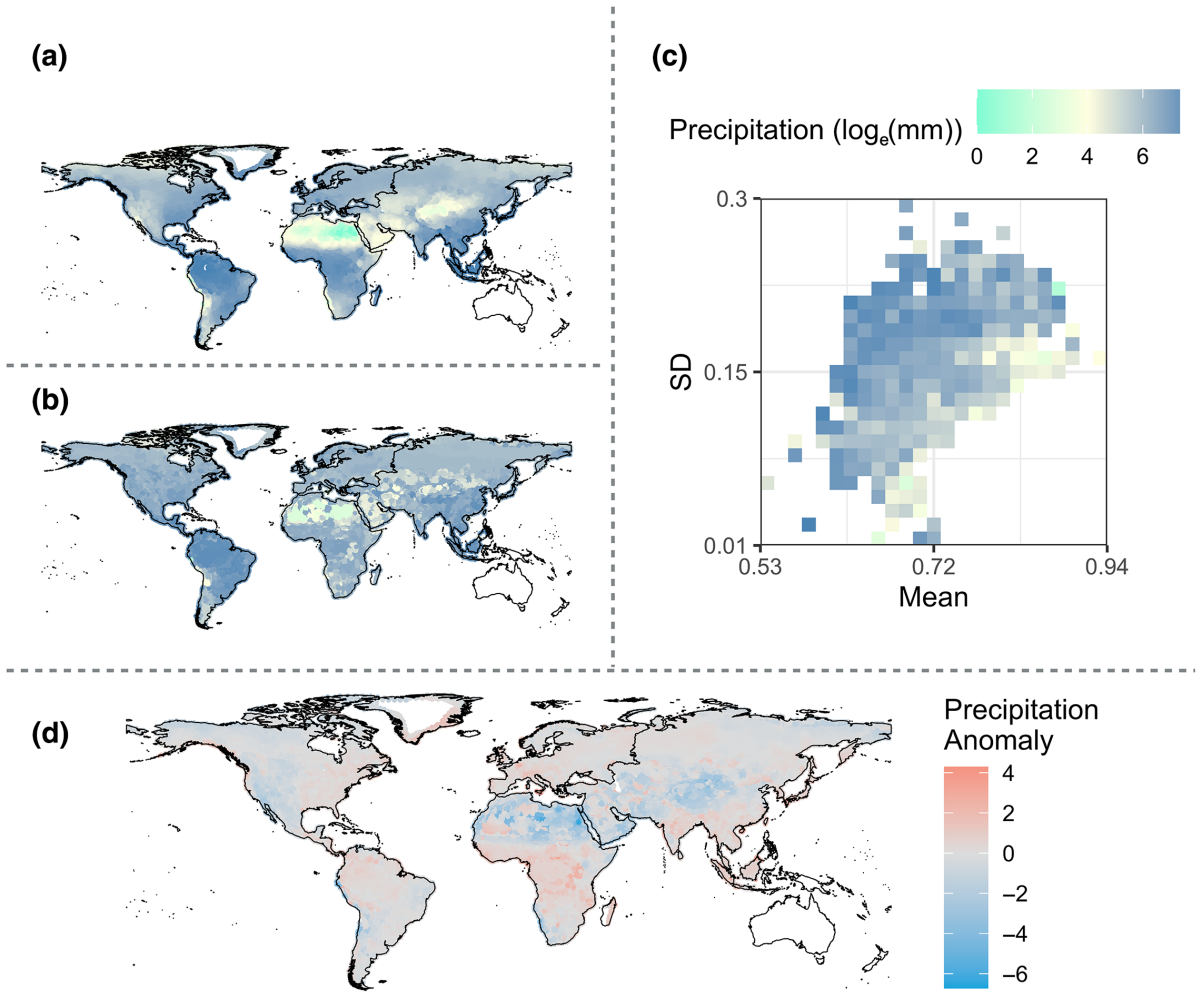
Ecometric model associated with global annual precipitation in millimeters natural log‐transformation (AP). (a) Observed AP (Fick & Hijmans, 2017). (b) Estimated global AP based on carnivoran RBL. (c) Ecometric space associated with carnivoran community RBL mean (*x*‐axis) and standard deviation (*y*‐axis), color‐coded based on the maximum likelihood estimate of AP in each grid cell. Each grid cell in the ecometric space indicates communities associated with a specific RBL mean and standard deviation. (d) Anomaly map representing geographic regions in which precipitation was overestimated or underestimated. An overestimate (blue) corresponds to a model‐based estimate that is wetter than the observed precipitation, while an underestimate (red) corresponds to a model‐based estimate that is drier than the observed precipitation.

**FIGURE 5:**
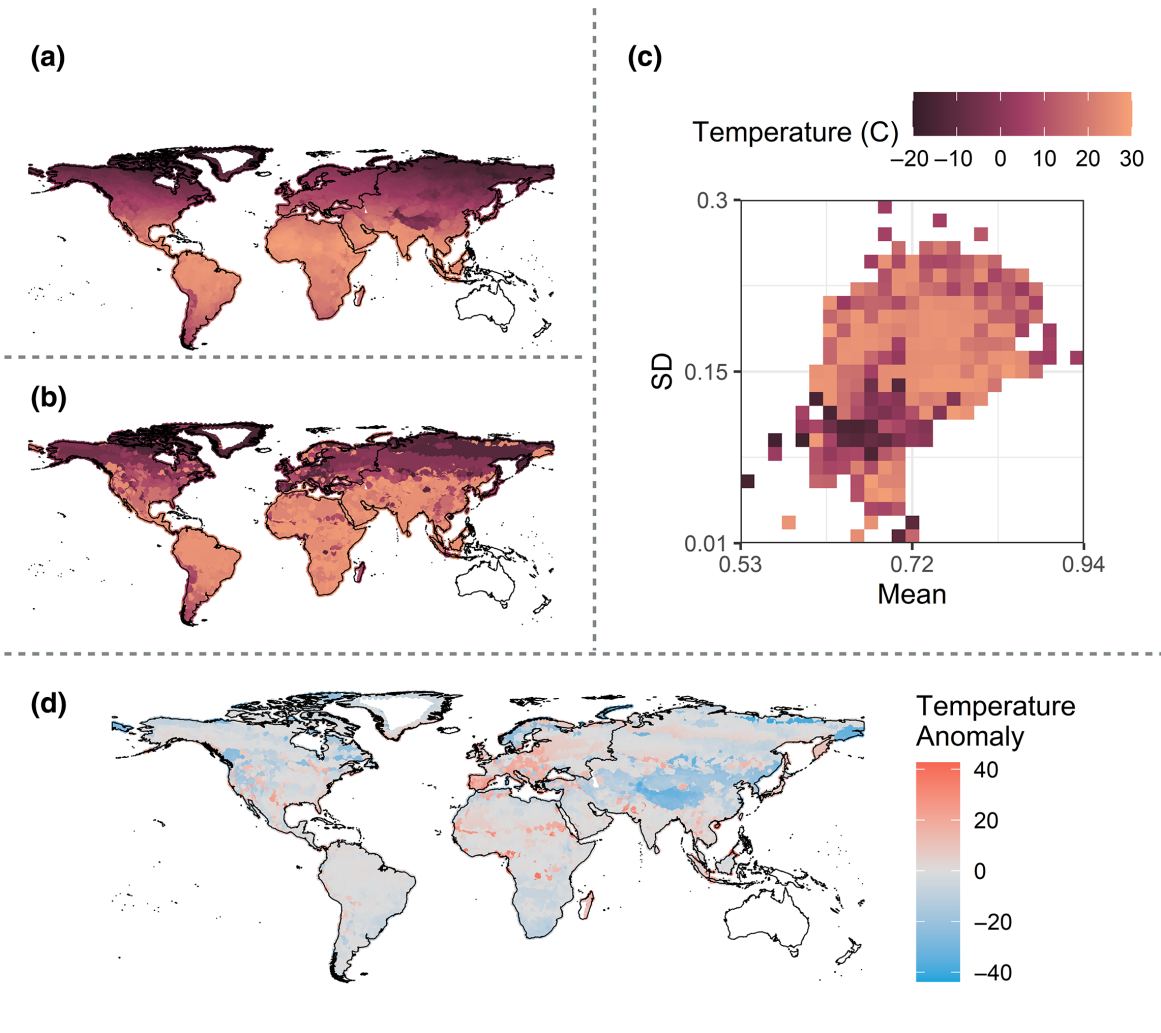
Ecometric model associated with global mean annual temperature (MAT). (a) Observed MAT (Fick & Hijmans, 2017). (b) Ecometric space associated with carnivoran community RBL mean (*x*‐axis) and standard deviation (*y*‐axis), color‐coded based on the maximum likelihood estimate of MAT in each grid cell. Each grid cell in the ecometric space indicates communities associated with a specific RBL mean and standard deviation. (c) Estimated global MAT based on carnivoran RBL. (d) Anomaly map representing geographic regions in which community RBL was overestimated or underestimated. An overestimate (blue) corresponds to a model‐based estimate that is warmer than the observed temperature, while an underestimate (red) corresponds to a model‐based estimate that is colder than the observed temperature.

Global patterns of observed precipitation were associated with our model estimates of expected precipitation (*R* ^2^ = .49, *p* < .001) and temperature (*R* ^2^ = .53, *p* < .001). The relationship associated with precipitation largely persisted across geographic regions, where the smallest amount of explained variance was in North America and Europe (*R* ^2^ = .45, *p* < .001 and *R* ^2^ = .46, *p* < .001, respectively) and the largest amount of explained variance was in Asia and South America (*R* ^2^ = .66, *p* < .001 and .64, *p* < .001, respectively) (Table 1; Figure 4; Figures S1 and S2). The strength of the relationship associated with temperature varied greatly by geographic region, such that North America was associated with a relatively high amount of explained variance (*R* ^2^ = .71, *p* < .001) and Africa was associated with a limited amount of explained variance (*R* ^2^ = .10, *p* < .001) (Table 1; Figure 5). Randomization procedures demonstrate that these relationships are not spurious (Figures S3–S6).

The highest community‐wide RBL variation occurred in South America, Africa, and Asia (standard deviations of 0.20, 0.14, 0.14, respectively), where the greatest amount of explained variance between observed and expected precipitation occurred (*R* ^2^ = .64, *p* < .001, *R* ^2^ = .59, *p* < .001, *R* ^2^ = .66, *p* < .001, respectively) (Table 1). In South America specifically, the RBL ratios ranged from 0.61 to 0.94, which is similar to values of the global sample, where global RBL ratios range from 0.53 to 0.94. In Europe, the precipitation model was less predictive (*R* ^2^ = .46, *p* < .001), and the RBL ratios ranged from 0.63 to 0.79 (Table 1).

The greatest amount of explained variance between observed and expected temperature occurred in North America, South America, and Asia (*R* ^2^ = .71, *p* < .001, *R* ^2^ = .67, *p* < .001, *R* ^2^ = .52, *p* < .001, respectively) (Table 1), geographic regions that are associated with the greatest variation in temperature (standard deviations of 123, 63, and 135, respectively). The lowest amount of overall explained variance occurred in Africa (*R* ^2^ = .10, *p* < .001) (Table 1), a geographic region that is associated with the least variation in temperature (standard deviation of 36).

The ecometric trait space associated with both annual precipitation and mean annual temperature was most strongly differentiated by trait standard deviation (Figures 4c and 5c). In wetter and hotter conditions, we find greater RBL standard deviation, indicating a wider variety of diets exploited by the community; whereas, in drier and more temperate conditions, we find less variation in the RBL trait, indicating a narrower breadth of dietary function. Both low and high mean RBL trait values are associated with an array of precipitation and temperature regimes; however, only wet conditions are associated with the lowest mean RBL values. Given that wetter habitats are associated with a high standard deviation and relatively low mean RBL trait values, this indicates that high precipitation areas can support carnivoran communities that include species with relatively minimal carnivorous habits.

### 3.4 Fossil carnivoran communities

**FIGURE 6:**
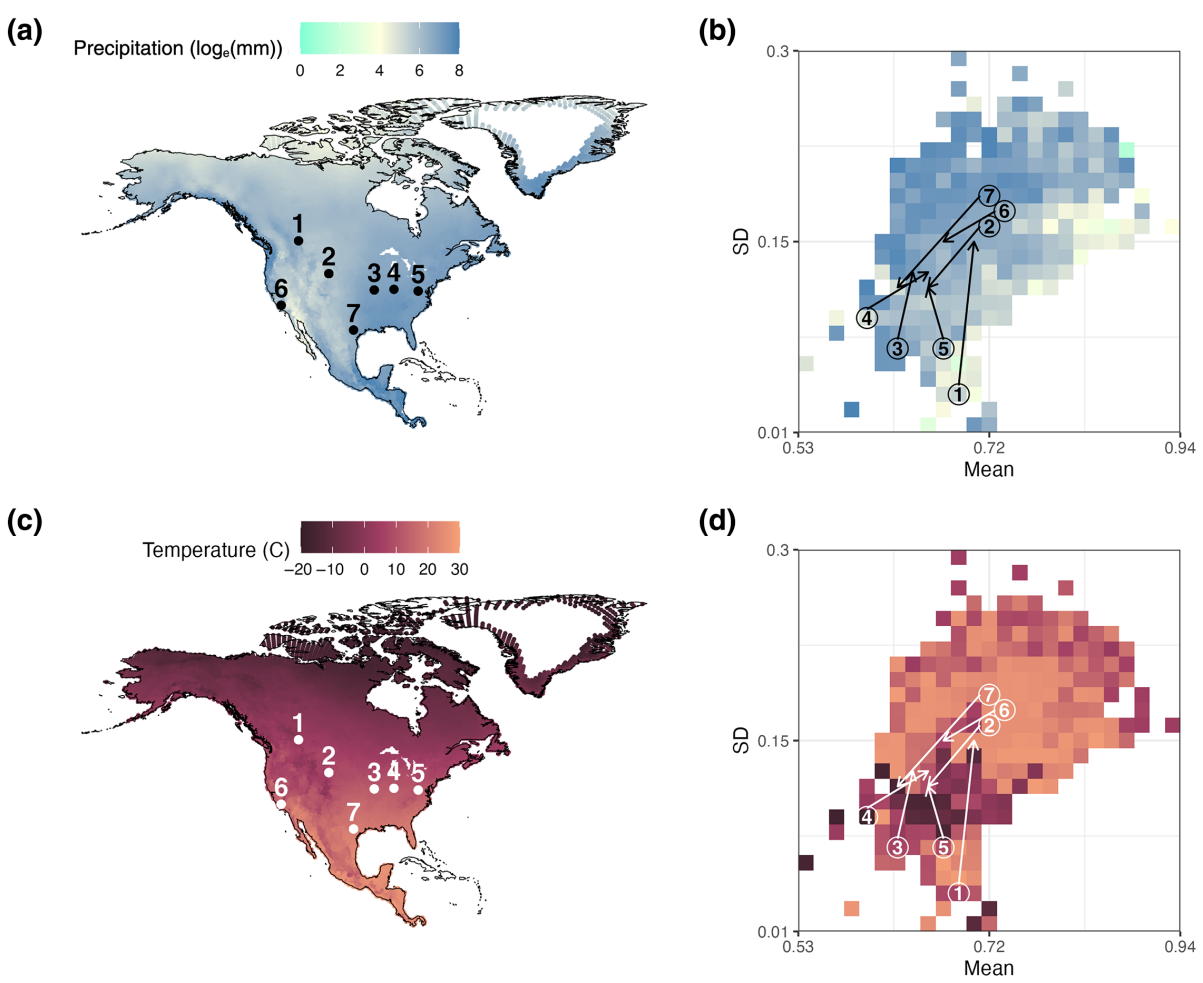
Fossil site paleo‐precipitation reconstructions for seven paleocommunities including, (1) January Cave, (2) Little Box Elder Cave, (3) Brynjulfson Cave 1, (4) Anderson Pit, (5) New Trout Cave, (6) McKittrick, and (7) Friesenhahn Cave. (a) Observed annual precipitation millimeters natural log‐transformation (AP). Fossil sites are numbered and indicated as black circles. (b) Trait turnover of RBL at each fossil site represented in ecometric space. (c) Observed mean annual temperature (MAT). Fossil sites are numbered and indicated as black circles. (d) Trait turnover of RBL at each fossil site represented in ecometric space. Ecometric space is based on the RBL and maximum likelihood estimate of AP, where each grid cell represents communities that display a given RBL mean and standard deviation and are color‐coded based on the maximum likelihood estimate of AP or MAT. Paleocommunities are represented in the ecometric space as numbered circles and arrows indicate the ecometric space inhabited by the modern communities located in the same geographic location.

We estimated paleoclimate conditions for North American fossil sites associated with the Last Glacial Maximum (Table 2; Figure 6). McKittrick, Little Box Elder Cave, and Friesenhahn Cave were all predicted to have been wetter (by 211, 386, and 590 mm, respectively) and hotter (by 9.02, 21.03, and 3.31°C, respectively) than modern conditions (Table 2). Over this time, McKittrick and Little Box Elder Cave shifted from predominantly forested habitats (coniferous and deciduous) to modern grasslands (Little Box Elder Cave Anderson, 1968; Long, 1971; McKay, 2008) or xeromorphic shrublands (McKittrick; Jefferson, 1991), while Friesenhahn Cave has primarily remained a grassland (Graham, 1976; Hall & Valastro Jr, 1995). The mean and standard deviation of the RBL trait also decreased at these sites as the habitats shifted to drier conditions. These changes were associated with a loss of hypercarnivorous species including saber‐toothed cats (*Smilodon fatalis*) from Friesenhahn Cave and McKittrick, scimitar‐toothed cats (*Homotherium serum*) from Friesenhahn Cave, and American lions (*Panthera atrox*), mountain lions (*Puma concolor*), and gray wolves (*Canis lupus*) from Little Box Elder Cave.

**TABLE 2:** Results of the paleoclimate reconstructions.

Site name	Age (KYA)	Fossil RBL (σ)	Modern RBL (σ)	Paleo habitat	Modern habitat	Predicted AP (mm)	Modern AP (mm)	Predicted MAT (C)	Modern MAT (C)
Friesenhahn Cave, TX	20–10^1^	0.74 (0.19)	0.64 (0.12)	Grassland^1,2^	Grassland	1434.39	844	22.91	19.6
Little Box Elder Cave, WY	24–10^3,4^	0.73 (0.16)	0.66 (0.12)	Conifer, boreal^3,4,5^	Grassland	810.61	425	24.53	3.5
New Trout Cave, WV	31.1–16.8^6^	0.68 (0.07)	0.67 (0.12)	Boreal, deciduous^7,8^	Cold deciduous	623.98	1003	8.49	9.6
Brynjulfson Cave, MO	34.6–9.4^9^	0.64 (0.08)	0.66 (0.12)	Boreal^8^	Cold deciduous	815.37	980	6.58	12.7
January Cave, AB	35–23.1^10^	0.70 (0.04)	0.72 (0.16)	Conifer, boreal, tundra^10^	Subpolar evergreen	216.74	572	6.77	1.6
Anderson Pit, IN	40–10^11^	0.61 (0.10)	0.66 (0.14)	Cold deciduous^12^	Cold deciduous	135.33	1150	−16.7	11.9
McKittrick, CA	40–10^13^	0.75 (0.18)	0.69 (0.16)	Open deciduous^13^	Xeromorphic shrubland	512.67	302	25.12	16.1

*Note*: Here we are including the site name, the site location, and approximate age in thousands of years ago (KYA), as well as the mean relative blade length (RBL) and standard deviation (σ) associated with the fossil and modern carnivoran communities from this site, and the predicted mean annual temperature (MAT) in Celsius (C) and annual precipitation (AP) in millimeters (mm) from the paleosite along with the modern MAT and AP for each site. Unless indicated otherwise, the site information (site name, age, and paleohabitat) has been compiled by Polly et al. (2017). See Appendix S2 for complete site data. Citations: ^1^Graham, 1976; ^2^Hall & Valastro Jr, 1995; ^3^Anderson, 1968; ^4^Long, 1971; ^5^McKay, 2008; ^6^Grady, 1986; ^7^Mead & Grady, 1996; ^8^Overpeck et al., 1992; ^9^Parmalee & Oesch, 1972; ^10^Burns, 1991; ^11^Richards, 1972; ^12^Smith & Polly, 2013; ^13^Jefferson, 1991.

New Trout, Brynjulfson, and January Caves were all predicted to have been drier (by 379, 165, and 355 mm, respectively) than modern conditions, with relatively stable temperatures, where modern conditions were predicted to have been marginally warmer in New Trout and Brynjulfson Caves (by 1.11 and 6.12°C, respectively) and marginally cooler at the January Cave site (by 5.17°C) (Table 2). These estimations align with expectations as New Trout and Brynjulfson Caves have likely shifted from boreal forests to cold deciduous forests (Mead & Grady, 1996; Overpeck et al., 1992) and January Cave has likely shifted from coniferous tundra to a subpolar evergreen habitat (Burns, 1991). The community‐wide mean RBL for these sites remained roughly constant over time, whereas the standard deviation increased as the conditions became wetter. Over time, the New Trout and Brynjulson Cave communities lost obligate carnivores (Table S1), including dire wolves (*Canis dirus*) and bobcats (*Lynx rufus*) but gained mid‐sized mesocarnivores, such as gray foxes (*Urocyon cinereoargenteus*) and eastern spotted skunks (*Spilogale putorius*). Likewise, January Cave gained both obligate carnivores, such as bobcats (*Lynx rufus*) and Canada lynx (*Lynx canadensis*), as well as dietary generalists, including American black bears (*Ursus americanus*) and striped skunks (*Mephitis mephitis*).

Anderson Pit is predicted to have been drier (1015 mm) and colder (28.6°C) than modern conditions (Table 2). Limited information is available about the paleohabitat associated with this site, although it may have been similar to the present deciduous forest associated with the modern location (Smith & Polly, 2013). The mean and standard deviation of the RBL trait increased at this site as this community gained obligate carnivores, such as bobcats (*Lynx rufus*), and mid‐sized generalists, such as red foxes (*Vulpes vulpes*).

## 4 DISCUSSION

Carnivoran dietary morphology represented by lower carnassial relative blade length (RBL) at the community level is associated with the climates in which carnivoran communities occur; thus, RBL provides some information about the climate inhabited by carnivorans (Figures 4 and 5). These ecometric models can be used to estimate paleoclimate, and the corresponding ecometric space provides a way to assess and visualize community‐level functional trait shifts. Given the variety of habitats and geographic regions that carnivoran communities occupy, the variation in their dietary niches, and the extensive preservation of lower carnassial teeth in the fossil record, carnivoran dietary traits are good candidates to form the basis of ecometric models.

### 4.1 Carnivoran diets

Carnivoran dental traits are related to dietary diversity and the percent of vertebrate prey in the diet. In our preliminary analyses, species whose diets are more strongly associated with vertebrate prey, display higher RBL values and those with more diverse diets, display lower values (Figure 1), relationships that each explained roughly 14% of the variation in species‐level RBL traits. This percent of explained variation is due, in part, to the flexibility in many carnivoran diets. Even species with limited dietary diversity may consume items outside of their normal habits as opportunity or need arises (Cruz et al., 2022; Metz et al., 2024; Middleton et al., 2021). Diets may also shift in response to changes in resource availability or community composition. For example, the loss of apex predators may change the resource acquisition behaviors of the mesocarnivores in a community (Karlin, 2017; Prugh et al., 2009). Regardless of this dietary flexibility, the RBL trait provides a proxy for diet, even when dietary behavior is not observable (e.g., paleocommunities).

### 4.2 Variations of traits and climates

We found greater community‐wide trait variation in areas with higher precipitation and temperature, where there is a greater variety of dietary items. For example, a community located in the Amazonian rainforest had 46% more trait variation as one located in the Saharan desert (*σ* = 0.24 and 0.15, respectively, Figure 3b). The diet composition of these communities varied in association with our expectations, where the diets of the desert community were primarily composed of vertebrate prey, with minimal deviations ($\bar{x}=82\%$ vertebrate prey; *σ* = 17) and the diet of the rainforest community was less closely tied to strictly carnivorous behaviors ($\bar{x}=63\%$ vertebrate prey; *σ* = 41%). Notably, both communities were composed of eleven carnivoran species, suggesting that this increase in trait variation is not simply a function of increased species richness within the rainforest. In fact, although the highest levels of annual precipitation are associated with South America, the greatest species richness is associated with Africa. However, Africa (x̄ = 14.41 species; *σ* = 6.56) and South America (x̄ = 13.06 species; *σ* = 4.07) have similar community species richness, suggesting that there is no systematic difference between the richness of these regions.

The ecometric relationship between dietary traits described here differs from the relationship associated with carnivoran locomotor traits. While Polly (2010) found that carnivoran locomotor traits were related to temperature, but not precipitation, we found a relationship of observed and expected environments constructed using carnivoran dietary traits for both precipitation and temperature. Similarly, in an ecometric analysis focused on overall carnivoran cranial shape, a trait which is also indicative of carnivoran diet, Meloro and Sansalone (2022) also found a relationship associated with both precipitation and mean annual temperature. Tseng & Flynn (2018) showed that herbivorous carnivorans were more common in areas with high precipitation and suggested an ecomorphological relationship where these species were more arboreal. Carnivoran postcranial morphology has been linked with diet (Iwaniuk et al., 1999), where carnivoran species with more herbivorous diets have been associated with arboreal locomotor styles (Dumont et al., 2016; McNab, 1995). Given that carnivoran locomotor traits have been less effective at predicting patterns of precipitation than the dietary traits presented here, we suggest that the increased RBL trait variation in high precipitation areas is likely related to the greater biodiversity in these areas, allowing for wider dietary opportunity. Notably, Polly (2010) also identified vegetation cover as a major factor associated with carnivoran locomotor traits, a factor that is also likely to be tied to dietary opportunity. Future studies should investigate the relationship between carnivoran dietary traits and vegetation cover as well as the potential predictive power of an integrative model incorporating both carnivoran dietary and locomotor traits.

Interestingly, because of the relationship with precipitation trends, the carnivoran dietary ecometric relationship is similar to the dietary ecometric trends of small herbivorous mammals (rodents and lagomorphs; Schap et al., 2021, Schap et al., 2024), hoofed mammals (artiodactyls and perissodactyls; Eronen, Puolamäki, et al., 2010; Fortelius et al., 2002; Fortelius et al., 2016; Short et al., 2021; Žliobaite et al., 2018), and the combined communities of small and hoofed herbivorous mammals (Short et al., 2021). Dental traits of small herbivorous mammals (Schap et al., 2021; Schap et al., 2024) as well as ungulates and primates (Oksanen et al., 2019) have also been linked to temperature in North America. In small mammals and hoofed mammals, tooth crown height (i.e., hypsodonty) was used as a climate proxy (Damuth & Janis, 2011; MacFadden, 1997; Stirton, 1947; Strömberg, 2002; Webb, 1977), where a higher crown is indicative of the airborne grit and dust that is consumed in an open, arid environment (Damuth & Janis, 2011; Damuth & Janis, 2014; Janis, 1988; Jardine et al., 2012; Jernvall & Fortelius, 2002; Semprebon et al., 2019; Williams & Kay, 2001), as well as the silica present in arid‐adapted plants (Erickson, 2014; Merceron et al., 2016; Strömberg, 2002). While hypsodonty reflects the tooth's ability to withstand abrasive dietary materials (Damuth & Janis, 2011; Kaiser et al., 2013), the carnivoran tooth morphology examined here is instead related to the relative ability to either slice or grind dietary items (Friscia et al., 2007; Tarquini et al., 2020; Van Valkenburgh, 1989). A lower carnassial tooth with a well‐developed trigonid blade allows for a scissor‐like slicing motion, which is highly effective for hypercarnivorous diets, whereas a lower carnassial tooth with more grinding space associated with a larger talonid basin allows an animal to process a variety of dietary items more strongly associated with frugivory or herbivory (Davies et al., 2007; Friscia et al., 2007; Van Valkenburgh, 1989). Therefore, the carnivoran dental morphology measured here is reflective of the vast dietary breadth represented by the order, which provides a different type of information than the dietary traits of strictly herbivorous guilds. While an herbivorous diet is intricately tied to primary productivity, the diet of a carnivoran community is reflective of the dietary opportunity available in the ecosystem and therefore may indicate the biodiversity of the area. Carnivoran communities located in less biodiverse regions may have fewer dietary options (e.g., exclusively small vertebrate prey) and therefore display reduced dietary breadths, whereas communities located in highly biodiverse regions may display wider dietary breadths associated with the varied dietary options. The fact that dietary ecometrics are strongly or moderately related to precipitation for both herbivorous and carnivorous groups suggests that this may be a useful relationship for other guilds in the future.

### 4.3 Carnivoran range loss

The geographic ranges inhabited by carnivoran species have shifted dramatically over the past century, leading to range contractions in many carnivoran species and range expansions in certain mesocarnivores (Di Minin et al., 2016; Fernández‐Sepúlveda & Martín, 2022; Ripple et al., 2014). In an evaluation of carnivoran locomotor ecometric models, Polly & Head (2015) noted the tremendous loss of functional trait diversity that has occurred across carnivoran communities during the Anthropocene and the pronounced effects on the fit of these models related to carnivoran extirpations. Conversely, our model successfully recovers RBL as an ecometric even with drastic recent losses and shifts within carnivoran ranges, indicating that partial disassembly of carnivoran communities has not completely removed the signal of this trait‐environment relationship. These results follow the findings of Polly and Sarwar (2014), which indicate that species losses representing less than 25% of fauna may add uncertainty to ecometric models but do not influence the overall proportion of explained variance detected by those models. Future studies should evaluate carnivoran dietary ecometric models over the past several centuries, with a particular interest in whether the strength of these models improves when constructed using historic geographic range data.

### 4.4 Geographic and continental trends

We document a relationship between carnivoran dental traits and climatic variables, where global patterns of observed precipitation and temperature corresponded with our model estimates (*R* ^2^ = .49 and *R* ^2^ = .53, respectively). These trends largely persisted across geographic regions; however, we did detect regional variations in the strength of these relationships (Table 1; Figures S1 and S2). This variation in model strength across geographic regions may suggest that future ecometric analyses may also benefit from this regional approach. This supports the recent recommendations of Wilson et al. (2023), which reported flaws in global ecometric models given the regional variations in traits and environments.

Europe had the weakest combined ecometric relationships for precipitation (*R* ^2^ = .46) and temperature (*R* ^2^ = .36) and also displayed the lowest mean RBL values (0.69) and the least amount of trait variation (*σ* = 0.11) as compared to the other continents. This may be related to the dramatic depletion of carnivorans across Europe, which has left the communities with predominantly generalist species (Dalerum, 2013; Dalerum et al., 2009; Wolf & Ripple, 2017). Although the model still functions within Europe, the fact that it is the least predictive geographic region may help to support the critical importance of carnivorans to their communities and it may portend poorly for the functionality of other communities in the face of future climate scenarios and the continued loss of other carnivores worldwide. This may also provide further support for the strength of this trait‐environment relationship and the utility of this ecometric model as a potential predictor of geographic regions at risk of ecological disruption as indicated by mismatches of the trait‐environment relationship.

Overall, the weakest relationship between observed and expected environments was associated with mean annual temperature in Africa. Notably, similar trends have been detected in ecometric models exploring the dietary traits of rodent communities (Schap et al., 2024). Africa is associated with a limited range of community‐wide RBL trait values, the hottest temperatures, and the narrowest range in temperature values compared to other continents. The combination of limited variation in both trait and environment may limit the performance of the ecometric model in this region. Comparatively, North America is associated with a wider spread of community‐wide RBL values and the greatest range in temperature and also showed the greatest amount of explained variance between observed and expected mean annual temperature (*R* ^2^ = .71).

The models performed moderately well on a global scale, although it under‐ and over‐predicted precipitation and temperature of certain geographic regions based on community‐wide RBL values (Figure 4c,d). For example, the topographic complexity associated with the Himalayan Mountains in Asia is associated with precipitation anomalies north and south of the range. South of the Himalayas, the model under‐predicts the precipitation of the region. Whereas, north of the Himalayas and into the Gobi Desert, the model has predicted higher than expected levels of annual precipitation. Likewise, the Himalayan Mountain region is associated with an under‐prediction of temperature. Regions of the Saharan Desert in Africa were also largely under‐predicted in the precipitation model, although certain regions within the Sahara Desert are over‐predicted. Precipitation was also under‐estimated in wet regions, like the southeastern United States. Such underestimations are consistent with those found in herbivore‐based ecometric research (Short et al., 2021; Short & Lawing, 2021) and are likely to be at least partially related to the maximum likelihood function used to estimate precipitation and temperature, which assigns the most likely data values within each trait bin and can therefore miss the most extreme values. These findings may also be related to the loss of omnivorous species like the eastern spotted skunk (*Spilogale putorius*; Gompper & Hackett, 2005). Omnivorous species are dietary generalists (relative to other species like obligate carnivores), and they are therefore well‐adapted to the wider variety of food items that are characteristic of higher‐precipitation areas. Thus, the loss of omnivores in the southeastern United States would reduce the prevalence of functional traits that are adapted to the region's wet conditions, leading to the observed ecometric underestimations of precipitation.

### 4.5 Paleoclimate predictions

Our model functioned as a paleoclimate indicator, although without known temperature and precipitation values from the paleoenvironments, it is difficult to fully validate these predictions. We ascertained the climatic trends of these regions by assessing our predicted paleoclimates in comparison to reconstructions of dominant vegetation communities for each of these sites. There are a variety of methods for reconstructing paleoclimates (e.g., isotope analyses, phytoliths, and pollen and faunal records), however, these techniques are site‐specific, and these data are not available for all fossil sites (Eiler, 2011; Rashid et al., 2019; Sun et al., 2020). Ecometric approaches to paleoenvironmental reconstruction can be robust given that these techniques can create global models that can be used to estimate a broad range of paleoenvironments given trait‐environment relationships and known community assemblages (Vermillion et al., 2018). Determining carnivoran assembly within paleo‐communities can provide a challenge given that many carnivoran species maintain small population sizes and may therefore be underrepresented in the fossil record. Carnivoran assembly may also be influenced by a variety of factors, including, but not limited to climate. For example, the loss of megaherbivores during the Late Pleistocene was likely to have had a direct influence on large apex carnivores (Galetti et al., 2018; Ripple et al., 2015). The paleoclimates predicted within our model largely conformed to our expectations given the vegetation communities supported in these regions. However, we encourage future studies to further validate these models by conducting site‐specific comparisons between ecometric reconstructions and other paleoenvironmental reconstruction techniques and by further considering changes beyond climate that might influence carnivoran community assembly. The current models were designed to address the major components of climate (temperature and precipitation), although a variety of additional factors may also influence these relationships including seasonality, fire regimes, and megaherbivore extinctions.

Each of the paleo‐sites included in our study was associated with the Last Glacial Maximum during the Pleistocene. This time period is notable given that it is associated with a massive loss of carnivoran species, particularly in Europe, South America, and North America, where all of our paleo‐sites were located (Dalerum, 2013; Dalerum et al., 2009; Wolf & Ripple, 2017). Given the extreme RBL values associated with felids, the loss of several felid species (e.g., *Smilodon fatalis* and *Homotherium serum*) is reflected in the reduction of mean RBL values associated with McKittrick, Friesenhahn Cave, and Little Box Elder Cave, fossil sites that had previously displayed the highest mean RBL values. Correspondingly, each of these sites also displayed the greatest loss in trait variation over this time, again reflecting the loss of community members with high RBL values. Our model therefore predicted a reduction in precipitation and temperature at these specific locations compared with modern communities, given that warmer, wetter communities were associated with higher community‐wide RBL mean and standard deviation. This appears to conform to expectations given that these habitats have likely either remained similar (Friesenhahn Cave; Graham, 1976; Hall & Valastro Jr, 1995) or have become more arid in modern times as these sites have shifted from forested habitats (Little Box Elder Cave and McKittrick; Anderson, 1968; Jefferson, 1991; Long, 1971; McKay, 2008) to modern grasslands (Little Box Elder Cave) and xeromorphic shrublands (McKittrick). This trait turnover from fossil sites to modern conditions may be informative as we look to future projections of species losses associated with carnivorans and the ways in which they may disrupt ecosystem functionality (Di Minin et al., 2016; Ripple et al., 2014; Wolf & Ripple, 2017).

The ability of this model to reconstruct paleotemperatures may have promising applications for the future given that previous dental ecometrics using herbivorous mammal taxa have had limited utility for temperature reconstructions (e.g., Fortelius et al., 2016; Oksanen et al., 2019). However, the fact that several of our sites are predicted to have been at least marginally warmer in the past (Friesenhahn Cave, Little Box Elder Cave, January Cave, McKittrick) may suggest that our precipitation predictions are robust.

### 4.6 Conclusions

In conclusion, we show that community‐level carnivoran dietary traits are indicative of climatic conditions both worldwide and at a continental level and that the relationship between carnivoran dietary traits and environments can be used to hindcast paleoclimates. Ecometric techniques have previously been used to explore the dietary traits of ungulates, rodents, and lagomorphs (Eronen et al., 2010b; Fortelius et al., 2014; Schap et al., 2021; Žliobaite et al., 2018) as well as the locomotor traits of ungulates and carnivorans (Polly, 2010; Short et al., 2023; Short & Lawing, 2021), but this study represents the first attempt to explore carnivoran dietary traits in this framework. Developing an understanding of how functional traits relate to the environment can provide a powerful window into our expectations of future change, which may be of particular importance when examining a taxonomic group that provides a pivotal contribution to ecosystem function and is also on the precipice of large‐scale species losses (Dalerum, 2013; Prevosti & Pereira, 2014; Ripple et al., 2014; Roemer et al., 2009). Understanding the relationship between carnivoran communities and environmental conditions can provide a clearer understanding of the factors that influence community assembly in this group. These findings can inform paleoenvironmental reconstructions as well as the impending responses of carnivorans to future projections of climate change to help devise more effective conservation strategies moving forward.

## AUTHOR CONTRIBUTIONS

**Leila Siciliano‐Martina:** Conceptualization (equal); data curation (lead); formal analysis (lead); investigation (lead); project administration (lead); visualization (equal); writing – original draft (lead); writing – review and editing (lead). **Jenny L. McGuire:** Conceptualization (equal); funding acquisition (lead); methodology (equal); writing – original draft (supporting); writing – review and editing (lead). **Maria A. Hurtado‐Materon:** Data curation (supporting); formal analysis (supporting); visualization (equal); writing – review and editing (equal). **Rachel A. Short:** Data curation (supporting); formal analysis (supporting); funding acquisition (supporting); methodology (equal); writing – review and editing (supporting). **Daniel A. Lauer:** Investigation (supporting); writing – review and editing (supporting). **Julia A. Schap:** Investigation (supporting); writing – review and editing (supporting). **Johannes Müller:** Funding acquisition (supporting); writing – review and editing (supporting). **Fredrick K. Manthi:** Funding acquisition (supporting); writing – review and editing (supporting). **Jason J. Head:** Funding acquisition (equal); writing – review and editing (equal). **A. Michelle Lawing:** Conceptualization (equal); funding acquisition (lead); methodology (equal); visualization (supporting); writing – original draft (supporting); writing – review and editing (supporting).

## CONFLICT OF INTEREST STATEMENT

We have no competing interests to declare.

## Supporting information


Appendix S1.

Appendix S2.

Data S1.

## Data Availability

Our complete RBL dataset (modern), RBL dataset (paleo), IUCN range maps, environmental data available at 50 km‐equidistant points, continental shape files, and the applicable R code are available from the Dryad Digital Repository: https://datadryad.org/stash/share/L5oHyuPlea9vh9ZegNiSxyDi6lauiZhY2y7RS‐rKIW8.
