# Supplementary material for: Ecometrics demonstrates that the functional dental traits of carnivoran communities are filtered by climate
Source: Ecol Evol. 2024 Oct 13;14(10):e70214. doi: 10.1002/ece3.70214 (PMC11471392; doi:10.1002/ece3.70214)
Supplement: Supplementary file 3 — Data S1. [file ECE3-14-e70214-s003.docx]

**Figure S1:** Global and continental ecometric space associated with carnivoran community RBL mean (x-axis) and standard deviation (y-axis), color-coded based on the maximum likelihood estimate of annual precipitation (AP) in each grid cell. Each grid cell in the ecometric space indicates communities associated with a specific RBL mean and standard deviation. A. Global ecometric space, B. Ecometric space of Africa, C. Ecometric space of Asia, D. Ecometric space of Europe, E. Ecometric space of North America, F. Ecometric space of South America.


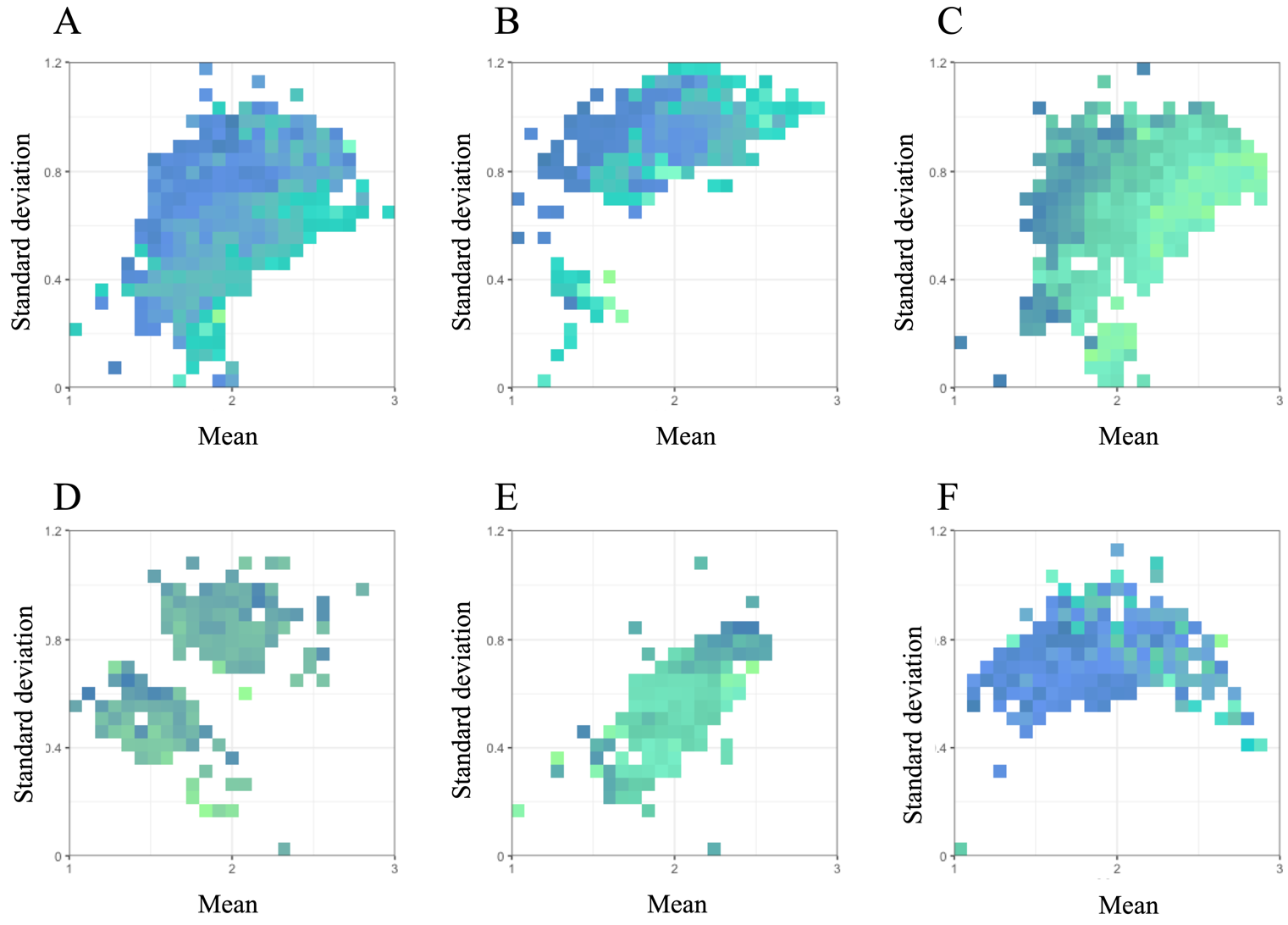


**Figure S2:** Global and continental ecometric space associated with carnivoran community RBL mean (x-axis) and standard deviation (y-axis), color-coded based on the maximum likelihood estimate of mean annual temperature (MAT) in each grid cell. Each grid cell in the ecometric space indicates communities associated with a specific RBL mean and standard deviation. A. Global ecometric space, B. Ecometric space of Africa, C. Ecometric space of Asia, D. Ecometric space of Europe, E. Ecometric space of North America, F. Ecometric space of South America.


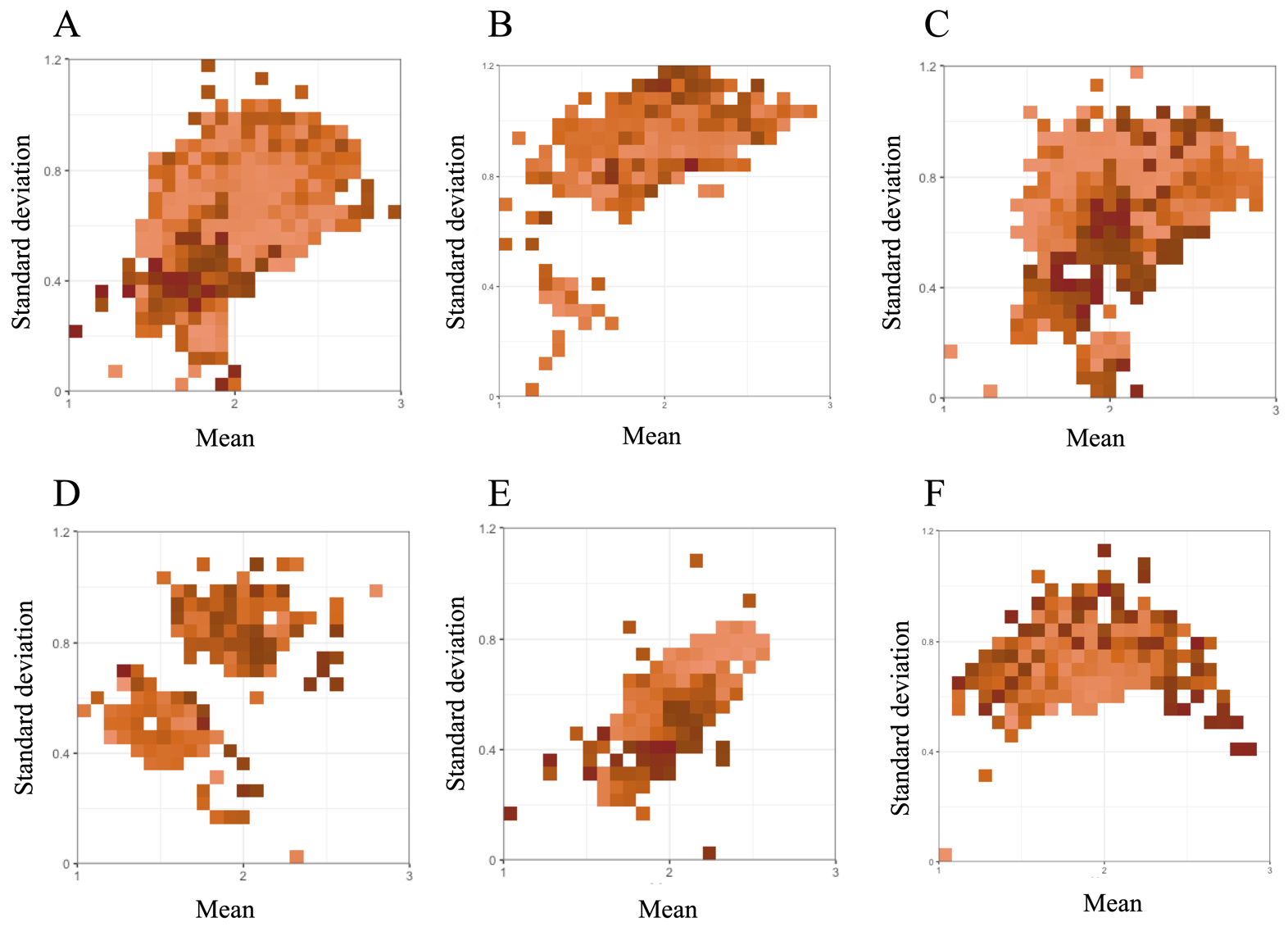


**Figure S3:** Sensitivity correlation plot associated with annual precipitation. A. Training data, B. Testing data.


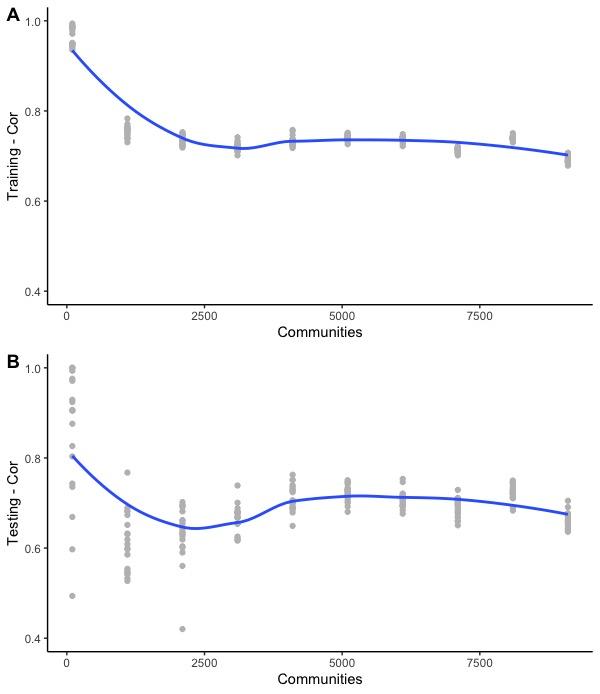


**Figure S4:** Sensitivity correlation plot associated with mean annual temperature. A. Training data, B. Testing data.


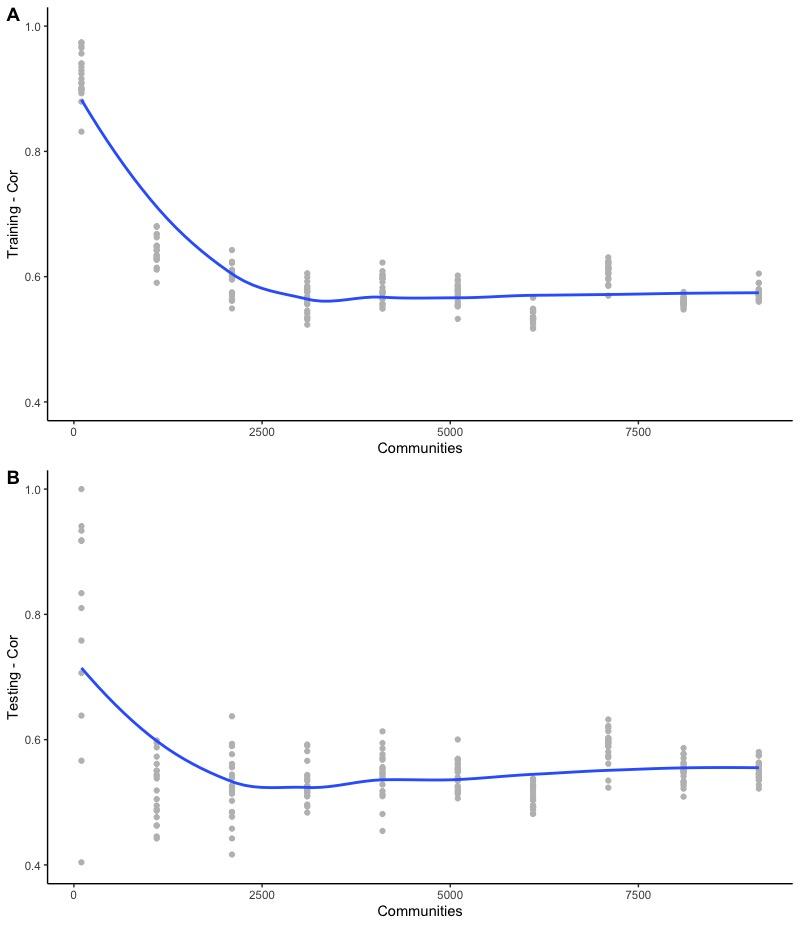


**Figure S5:** Sensitivity anomaly plot associated with annual precipitation. A. Training data, B. Testing data.


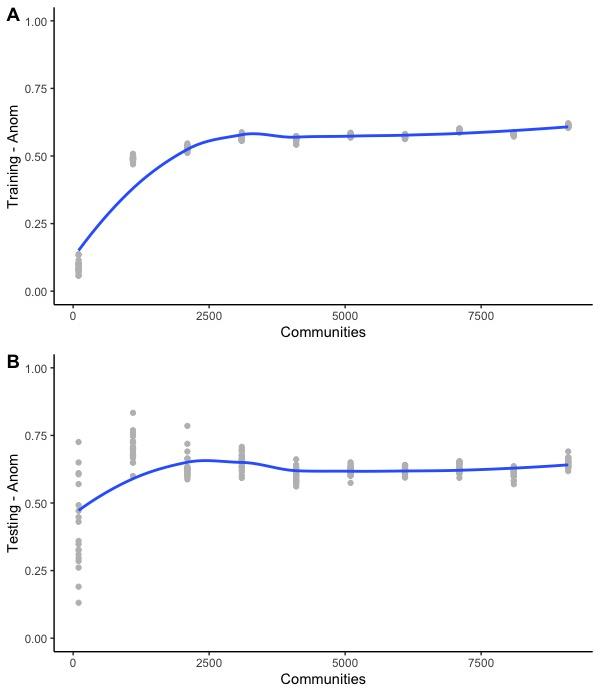


**Figure S6:** Sensitivity anomaly plot associated with mean annual temperature. A. Training data, B. Testing data.


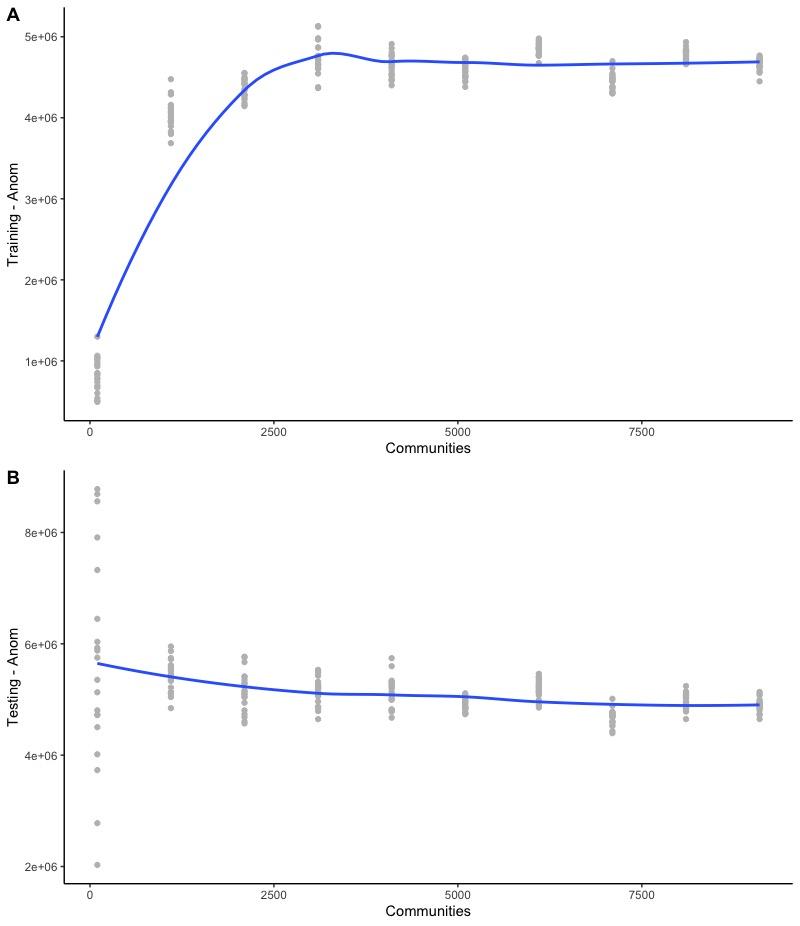


**Table S1**: Carnivoran species found in the fossil sites (gray columns) and their modern equivalents (white columns). Sites include Anderson Pit (AP), Brynjulfson Cave (BC), Friesenhahn Cave (FC), January Cave (JC), Little Box Elder Cave (LBEC), McKittrick (Mc), New Trout Cave (NTC).

| Family | Species | AP | AP | BC | BC | FC | FC | JC | JC | LBEC | LBEC | Mc | Mc | NTC | NTC |
| --- | --- | --- | --- | --- | --- | --- | --- | --- | --- | --- | --- | --- | --- | --- | --- |
| Canidae | *Canis*  *dirus* |  |  | X |  | X |  |  |  |  |  | X |  | X |  |
|  | *Canis*  *latrans* |  | X | X | X | X | X |  | X | X | X | X | X | X | X |
|  | *Canis*  *lupus* |  |  |  |  |  |  |  | X | X |  | X |  |  |  |
|  | *Urocyon*  *cinereoargenteus* | X | X | X | X | X | X |  |  |  |  |  | X |  | X |
|  | *Vulpes*  *macrotis* |  |  |  |  |  |  |  |  |  |  | X | X |  |  |
|  | *Vulpes*  *velox* |  |  |  |  |  |  |  |  |  | X |  |  |  |  |
|  | *Vulpes*  *vulpes* |  | X | X | X |  | X | X | X | X | X |  |  | X | X |
| Felidae | *Homotherium*  *serum* |  |  |  |  | X |  |  |  |  |  |  |  |  |  |
|  | *Lynx*  *canadensis* |  |  |  |  |  |  |  | X |  |  |  |  |  |  |
|  | *Lynx*  *rufus* |  | X |  | X | X | X |  | X | X | X | X | X |  | X |
|  | *Panthera*  *atrox* |  |  |  |  |  |  |  |  | X |  | X |  |  |  |
| Family | Species | AP | AP | BC | BC | FC | FC | JC | JC | LBEC | LBEC | Mc | Mc | NTC | NTC |
|  | *Puma*  *concolor* |  |  |  |  |  |  |  | X | X |  | X | X |  |  |
|  | *Smilodon*  *fatalis* |  |  |  |  | X |  |  |  |  |  | X |  |  |  |
| Mephitidae | *Conepatus*  *leuconotus* |  |  |  |  |  | X |  |  |  |  |  |  |  |  |
|  | *Mephitis*  *mephitis* |  |  | X | X | X | X |  | X | X | X | X | X |  | X |
|  | *Spilogale*  *putorius* |  |  | X | X |  |  |  |  | X | X | X |  |  | X |
| Mustelidae | *Gulo*  *gulo* |  |  |  |  |  |  |  |  | X |  |  |  |  |  |
|  | *Lontra*  *canadensis* |  | X | X | X |  | X |  | X |  | X |  | X |  | X |
|  | *Martes*  *americana* |  |  |  |  |  |  | X | X |  |  |  |  | X |  |
|  | *Martes*  *pennanti* |  |  | X |  |  |  |  |  |  |  |  |  | X | X |
|  | *Mustela*  *erminea* |  |  |  |  |  |  | X | X |  | X |  |  | X |  |
|  | *Mustela*  *frenata* |  | X | X | X |  | X | X | X | X | X | X | X | X | X |
|  | *Mustela*  *nigripes* |  |  |  |  |  |  |  | X | X | X |  |  |  |  |
|  | *Mustela*  *nivalis* |  |  |  |  |  |  | X | X |  |  |  |  | X | X |
| Family | Species | AP | AP | BC | BC | FC | FC | JC | JC | LBEC | LBEC | Mc | Mc | NTC | NTC |
|  | *Neovison*  *vison* | X | X | X | X |  |  |  | X |  | X |  |  | X |  |
|  | *Taxidea*  *taxus* |  |  |  | X |  | X |  | X | X | X | X | X | X |  |
| Procyonidae | *Bassariscus*  *astutus* |  |  |  |  |  | X |  |  |  |  |  |  |  |  |
|  | *Procyon*  *lotor* | X | X | X | X | X | X |  |  |  | X |  | X | X | X |
| Ursidae | *Arctodus*  *simus* |  |  |  |  | X |  |  |  | X |  | X |  |  |  |
|  | *Ursus*  *arctos* |  |  |  |  |  |  |  | X | X | X |  | X |  |  |
|  | *Ursus*  *americanus* | X | X | X | X | X | X |  | X |  | X | X |  | X | X |
